# Supplementary material for: PIF transcriptional regulators are required for rhythmic stomatal movements
Source: Nat Commun. 2024 May 29;15:4540. doi: 10.1038/s41467-024-48669-4 (PMC11137129; doi:10.1038/s41467-024-48669-4)
Supplement: Supplementary file 1 — Supplementary Information [file 41467_2024_48669_MOESM1_ESM.pdf]

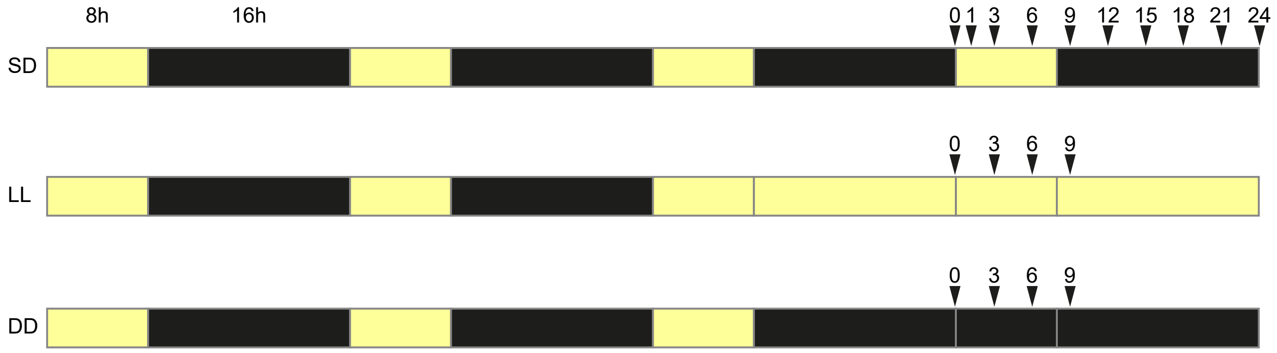

Supplementary Figure 1. Schematic diagram of the SD, LL, and DD growth regimes used. After vernalization, plates were transferred at the beginning of the day to short days (SD) (8h light + 16 h dark) for seedlings to germinate. Sampling in SD took place in the dark during the end of the third day (ZT=0), and in the light (ZT=1, 3, 6) or in the dark (ZT=9, 12, 15, 18, 21, 24) during the fourth day of growth. Continuous light (LL) samples correspond to time points taken in seedlings entrained in SD and then transferred to continuous light from the third night onward. Continuous dark (DD) samples correspond to time points taken in seedlings entrained in SD and then transferred to continuous dark from the third night onward. Yellow and black rectangles represent light and dark, respectively, and arrow heads indicate sampling time points.

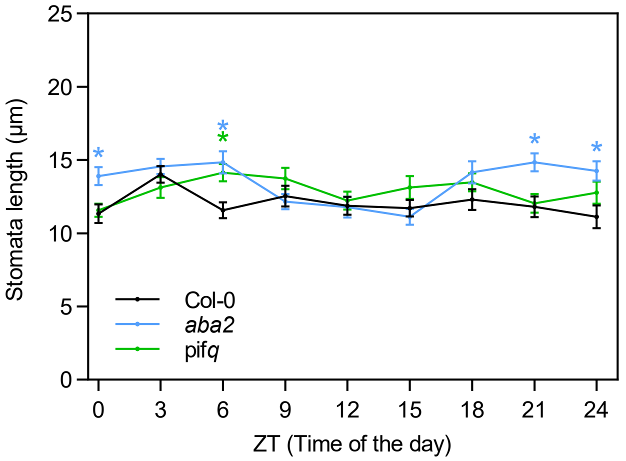

Supplementary Figure 2. Time course analysis of stomata length under SD. Time course analysis of stomata length in Col-0, *pifq*, and *aba2* cotyledons over 24 hours during the fourth day of seedling growth under SD conditions. Time points represent mean values ± SE. n=40 biologically independent samples. Statistical differences relative to Col-0 for each time point are indicated by an asterisk (Mann-Whitney test. *P*<0.05). Precise *P* values are provided in the Source Data file.

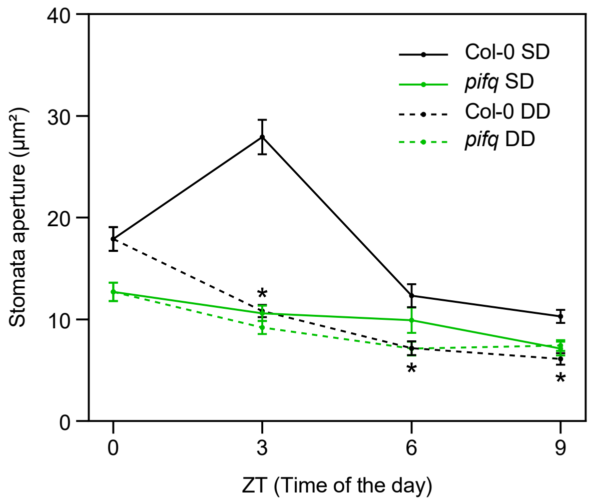

Supplementary Figure 3. Time course analysis of stomata aperture in Col-0 and *pifq* grown under DD. Seedlings were grown under short day (SD) conditions for 2 days, then at ZT8 of the third day they were either kept under SD or transferred to continuous dark (DD) (see SI Fig. 1 for a diagram of light treatments). Stomata pore measurements were performed during the fourth day at ZT0, 3, 6 and 9h and expressed as area. Time points represent mean values ± SE. n=51-60 biologically independent samples (precise n values for each genotype and time point are provided in the Source Data file). Statistical differences relative to Col-0 for each time point and condition are indicated by an asterisk (Mann-Whitney test. $P<0.05$). Precise P values are provided in the Source Data file.

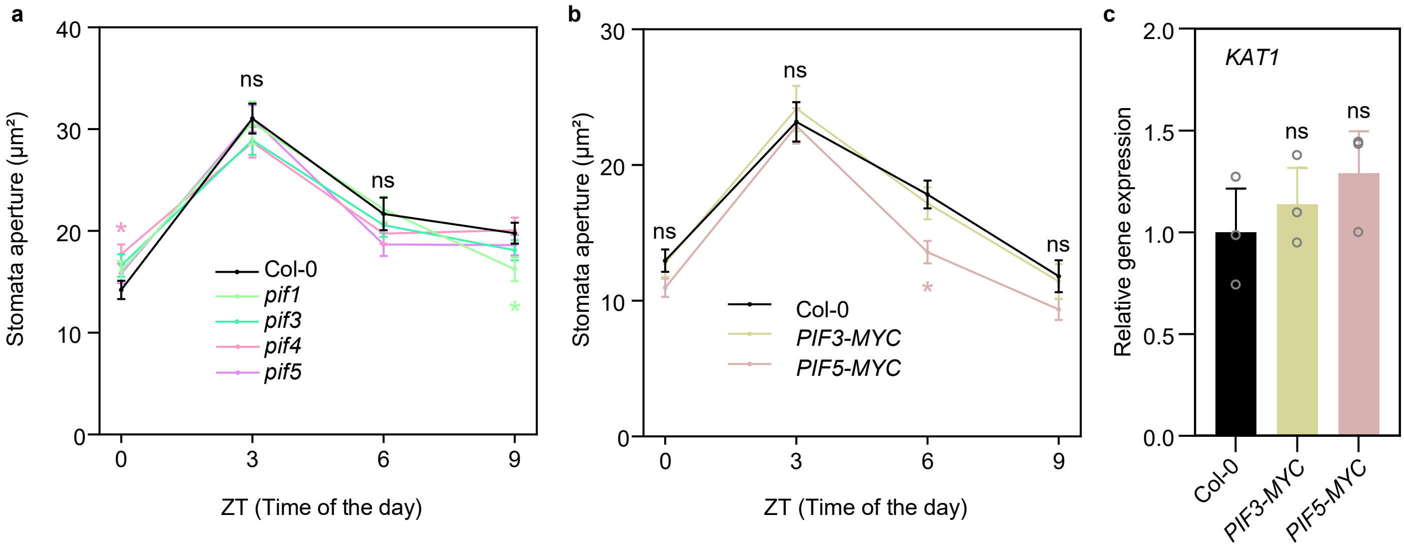

Supplementary Figure 4. Time course analysis of stomata aperture in *pif* single mutants and PIF overexpressing lines. Stomata aperture (expressed as area) in Col-0, *pif1, pif3, pif4,* and *pif5* (a) or Col-0, PIF3-OX, and PIF5-OX (b) grown under short days (SD). Stomata pore measurements were performed during the fourth day at ZT0, 3, 6 and 9h and expressed as area. Time points represent mean values ± SE. n=42-110 biologically independent samples (precise n values for each genotype and time point are provided in the Source Data file). Statistical differences relative to Col-0 for each time point and condition are indicated by an asterisk (Mann-Whitney test. P<0.05). Precise P values are provided in the Source Data file. (c) *KAT1* expression in Col-0 and PIF-OX lines at ZT0. Data are the means ± SE of biological triplicates (n = 3). n.s. not significant differences between Col-0 and PIF-OX samples (t-test; *, P < 0.05). Precise P values are provided in the Source Data file.

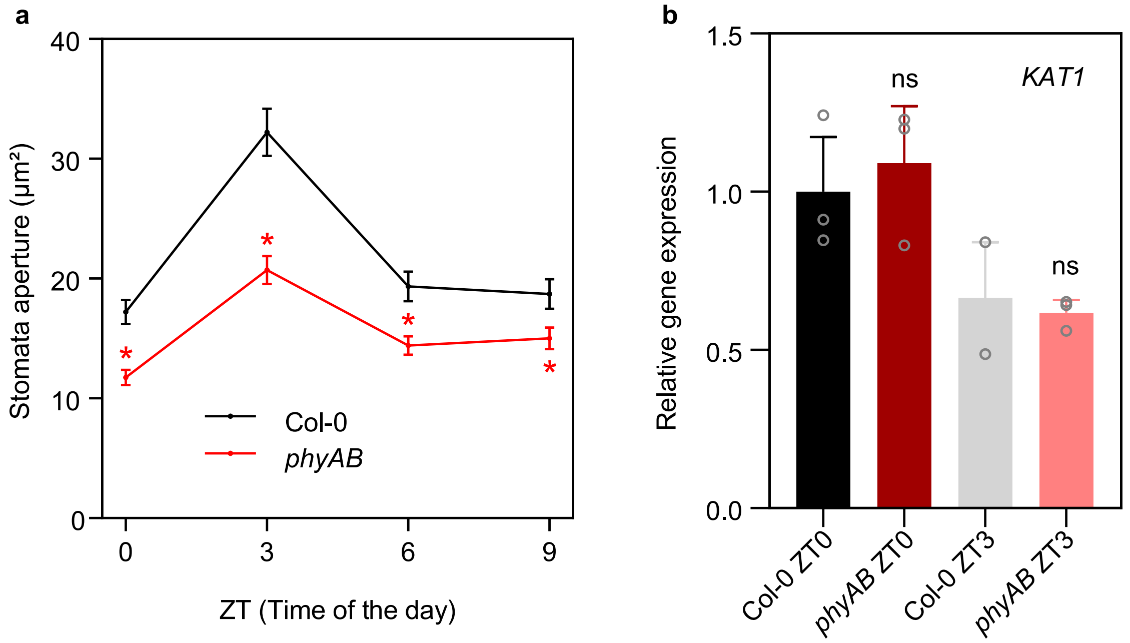

Supplementary Figure 5. Phytochrome regulation of stomata opening and *KAT1* expression in dark/light cycles. (a) Time course analysis of stomata aperture in Col-0 and *phyAB*, during the fourth day of seedlings grown under SD conditions. Time points represent mean values ± SE. n=50-97 biologically independent samples (precise n values for each genotype and time point are provided in the Source Data file). Statistical differences relative to Col-0 for each time point are indicated by an asterisk (Mann-Whitney test. $P < 0.05$). Precise P values are provided in the Source Data file. (b) *KAT1* expression in 3-day-old SD-grown Col-0 and *phyAB* seedlings at ZT0 and ZT3. Data are the means ± SE of biological triplicates (n = 3). Statistical differences between mean values at each time point were analyzed by Student *t*-test (P<0.05), and non-significant (n.s.) differences are indicated relative to Col-0.

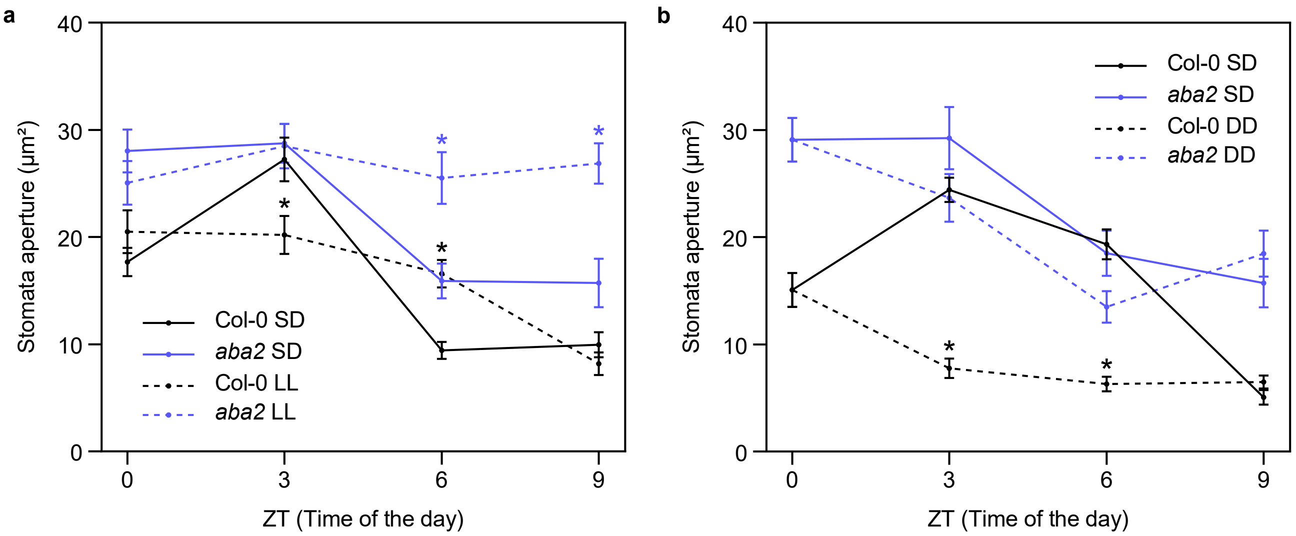

Supplementary Figure 6. Time course analysis of stomata aperture in Col-0 and *aba2* grown under LL or DD. Seedlings were grown under short days (SD) for 2 days, then at ZT8 of the third day they were either kept under SD or transferred to (a) continuous light (LL) or (b) continuous dark (DD). Stomata pore measurements were performed during the fourth day at ZT0, 3, 6 and 9h and expressed as area. Time points represent mean values ± SE. n=37-60 biologically independent samples (precise n values for each genotype and time point are provided in the Source Data file). (a, b) Statistical differences relative to Col-0 for each time point and condition are indicated by an asterisk (Mann-Whitney test. $P<0.05$). Precise P values are provided in the Source Data file.

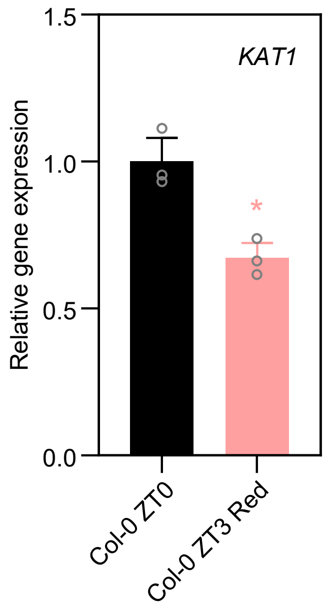

Supplementary Figure 7. *KAT1* expression in Col-0 after exposure to red light for 3h. *KAT1* expression in 3-day-old SD-grown Col-0 seedlings at ZT0 and ZT3. After growth for 3 days in control conditions, seedlings were exposed to 3h of red light (40 umols/m2.s) at ZT0 and samples were collected at ZT3. Data are the means ± SE of biological triplicates (n = 3). Statistical differences relative to Col-0 is indicated by an asterisk (t-test; *, P < 0.05). The precise *P* value is provided in the Source Data file.

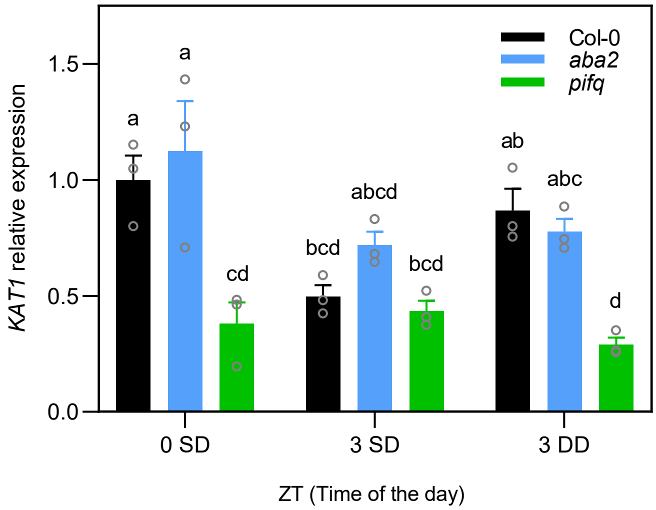

Supplementary Figure 8. *KAT1* expression in Col-0, *pifq*, and *aba2* seedlings under DD compared to SD. *KAT1* expression in Col-0, *pifq*, and *aba2* seedlings in 3-day-old SD- or DD-grown at ZT0 (common for SD and DD) and ZT3. Data are the means ± SE of biological triplicates (n = 3). Letters denote the statistically significant differences using 2-way Anova followed by posthoc Tukey's test (P < 0.05). Precise *P* values are provided in the Source Data file.

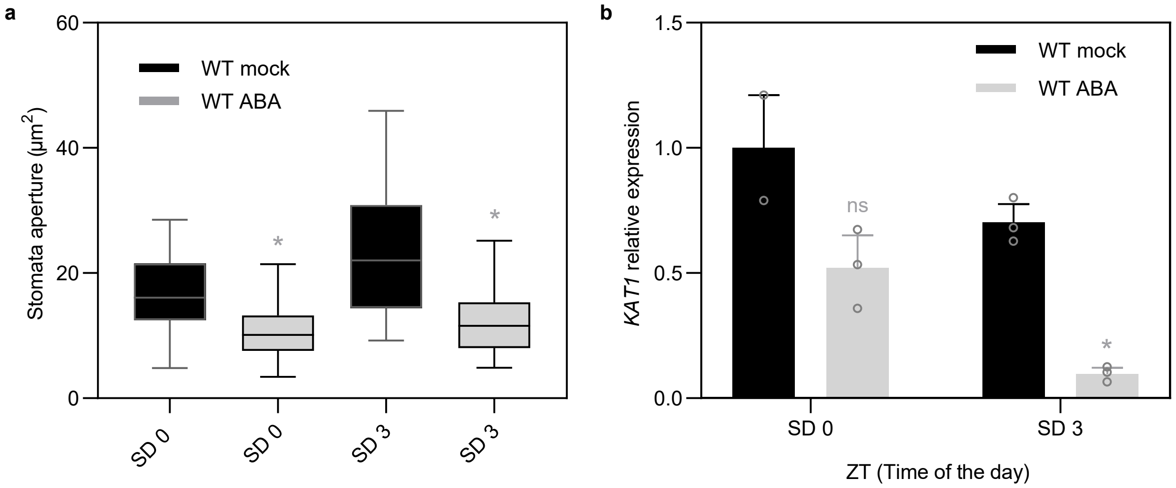

Supplementary Figure 9. Exogenously applied ABA represses *KAT1* expression and prevents stomata opening in dark/light cycles. (a) Stomata aperture in Col-0 wild-type seedlings (WT) treated with ABA (50 uM) or mock during the fourth day of growth under SD conditions at ZT0 and ZT3. n=47-77 biologically independent samples (precise n values for each genotype and time point are provided in the Source Data file). Statistical differences of ABA-treated samples relative to mock for each time point are indicated by an asterisk (Mann-Whitney test. $P < 0.05$). Precise *P* values are provided in the Source Data file. (b) *KAT1* expression in 3-day-old SD-grown Col-0 wild-type seedlings (WT) treated with ABA (50 uM) or mock at ZT0 and ZT3. Data are the means ± SE of biological triplicates (n = 3). Statistical differences between mean values at each time point were analyzed by Student *t*-test ($P<0.05$), and significant differences relative to mock are indicated with an asterisk. n.s., non-significant. Precise *P* values are provided in the Source Data file.

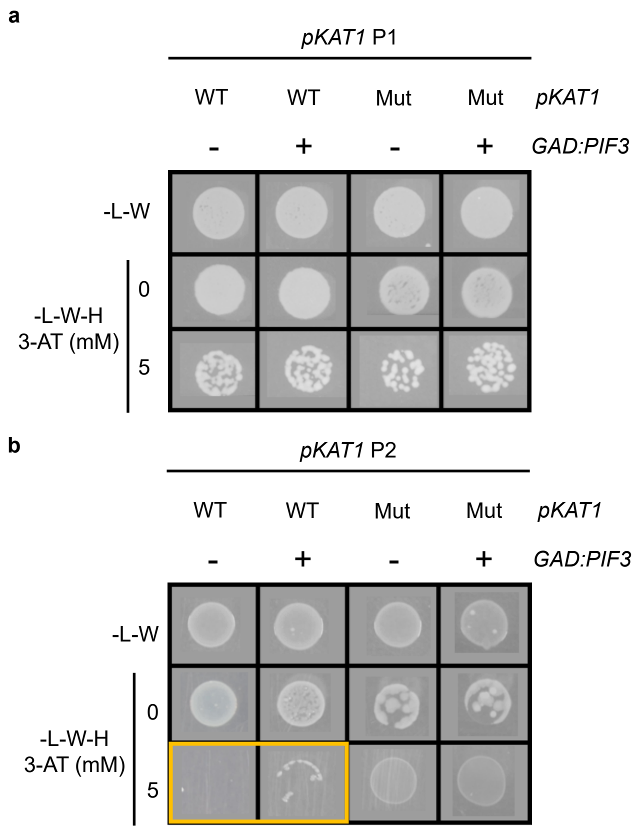

Supplementary Figure 10. PIF3 specifically binds to the P2 region of the *KAT1* promoter through the G-box motif. Yeast one hybrid assay with yeast strains containing either the P1 (a) or P2 (b) regions of the *KAT1* promoter (*pKAT1*) carrying the PIF binding elements G-box and PBE-box (*pKAT1* P1 WT), or G-box (*pKAT1* P2 WT) (Fig. 5a), or a mutated version in each element (*pKAT1* P1 Mut and *pKAT1* P2 Mut), which were mated to a strain containing the GAD-PIF3 construct. Diploid cells were grown on auxotrophic media with 3-AT. The experiments were repeated twice with similar results.

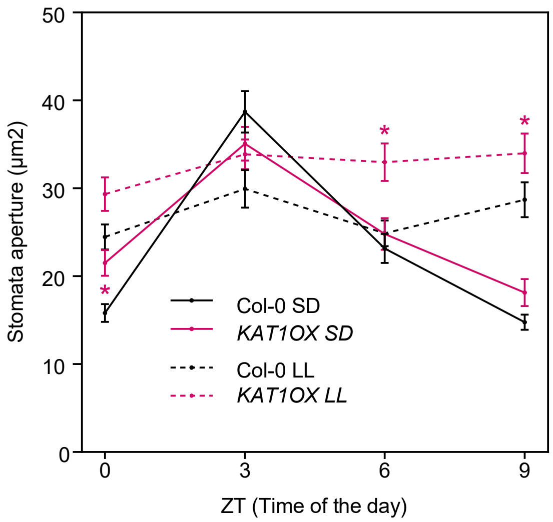

Supplementary Figure 11. Stomata dynamics of *KAT1OX* in LL. Time course analysis of stomata aperture (expressed as area) in Col-0 and *KAT1OX* grown under short days (SD) or SD transferred to continuous light (LL). Seedlings were grown under SD conditions for 2 days, then at ZT8 of the third day they were either kept under SD as a control (SD) or they were transferred to continuous white light (LL). Stomata measurements were performed during the fourth day at ZT0, 3, 6 and 9h. Time points represent mean values ± SE. n=55-113 biologically independent samples (precise n values for each genotype and time point are provided in the Source Data file). Statistical differences relative to Col-0 for each time and condition, are indicated by an asterisk (Mann-Whitney test. $P<0.05$). Precise P values are provided in the Source Data file.

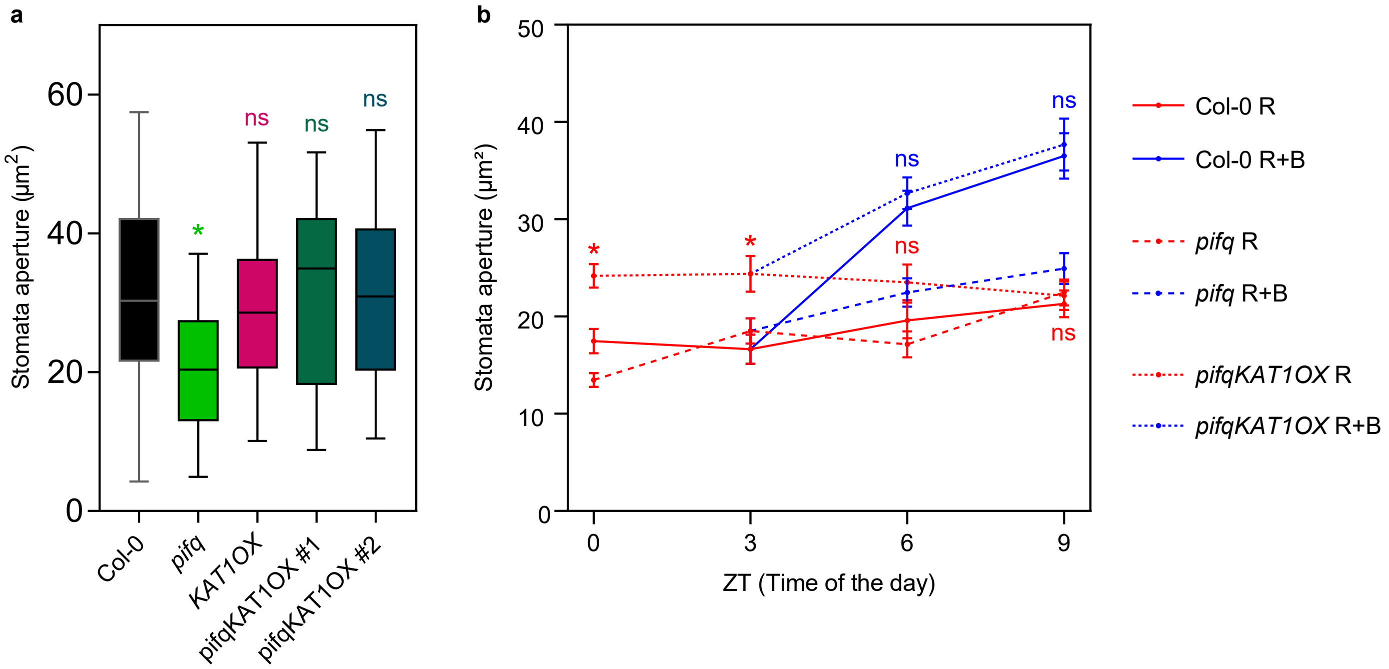

Supplementary Figure 12. *KAT1* overexpression restores stomatal dynamics of *pifq*. (a) Stomata aperture (expressed as area) in 3-day-old SD-grown Col-0, *pifq*, *KAT1OX*, and *pifqKAT1OX* at ZT3. n=48-76 biologically independent samples (precise n values for each genotype and time point are provided in the Source Data file). Statistical differences relative to Col-0 are indicated by an asterisk (Mann-Whitney test. $P<0.05$). Precise P values are provided in the Source Data file. (b) Time course analysis of stomata aperture in 3-day-old SD-grown Col-0, *pifq,* and *pifqKAT1OX* seedlings exposed to 3h of red light (40 umols/m2.s). In this background, blue light (10 umols/m2.s) was added for an additional 6h. Controls were kept in red light only for the whole duration of the experiment. Time points represent mean values ± SE. n=46-99 biologically independent samples (precise n values for each genotype and time point are provided in the Source Data file). Statistical differences relative to Col-0 for each time point and condition are indicated by an asterisk (Mann-Whitney test. $P<0.05$). Precise P values are provided in the Source Data file.

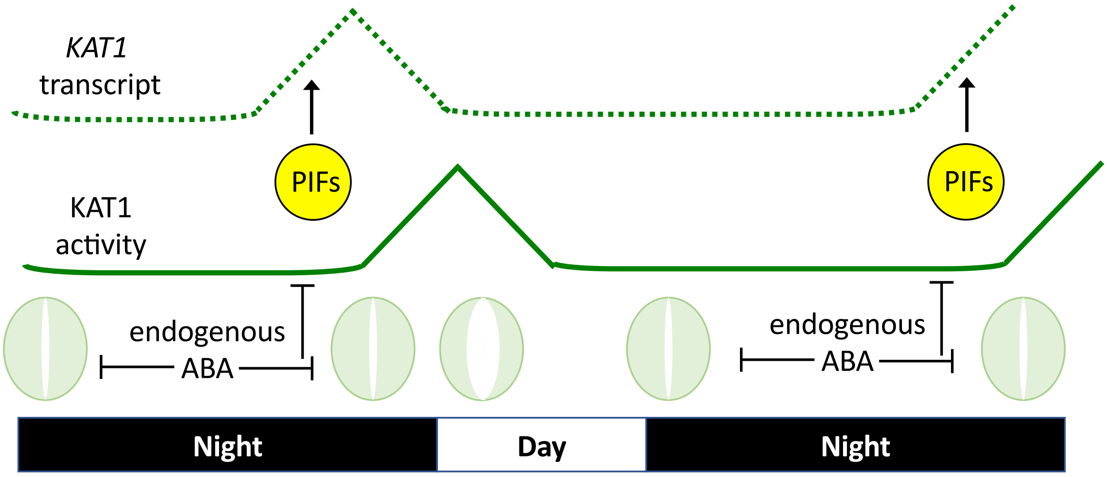

Supplementary Figure 13. Model of PIF and endogenous basal ABA interplay in the diurnal regulation of KAT1 in stomata dynamics. During the night in short days, PIFs progressively accumulate and induce *KAT1* expression at the end of the night. Endogenous ABA also accumulates at night and prevents activity of KAT1. At dawn, light induces ABA degradation and KAT1 can accumulate and promote stomata opening downstream of light-activated phot1 driving membrane hyperpolarization. Light also triggers phytochrome-mediated PIF degradation, effectively inhibiting *KAT1* overexpression.

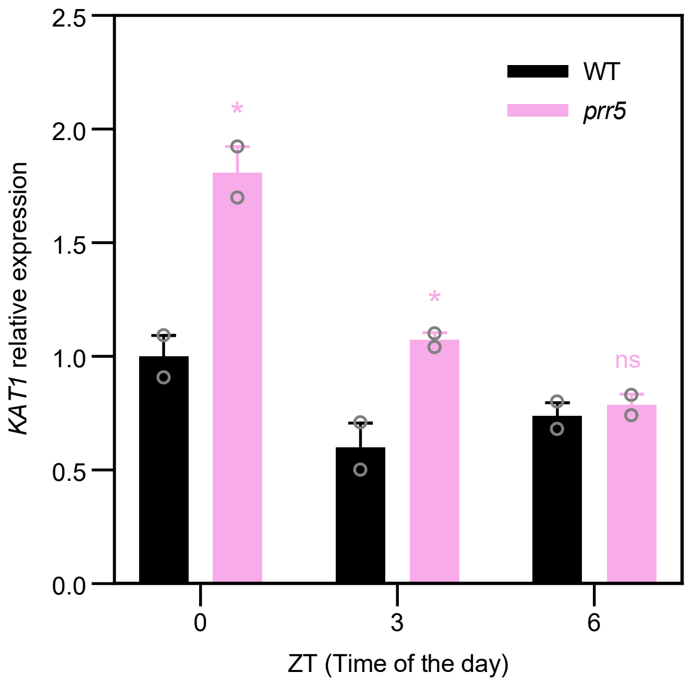

Supplementary Figure 14. *KAT1* expression in *prr5*. *KAT1* expression in 3-day-old SD-grown Col-0 and *prr5* seedlings at ZT0, ZT3 and ZT6. Data are the means ± SE of biological duplicates (n = 2). Statistical differences relative to Col-0 in each time point are indicated by an asterisk (t-test; *, $P < 0.05$). Precise *P* values are provided in the Source Data file.

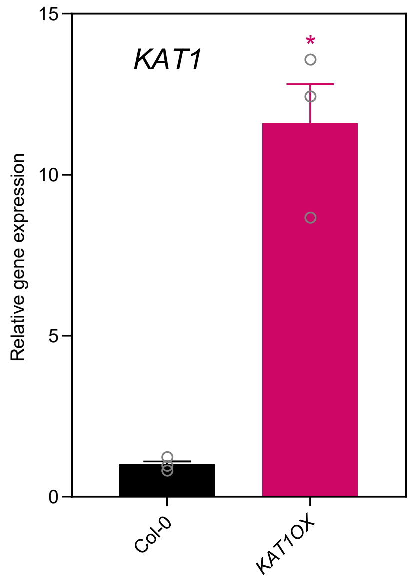

Supplementary Figure 15. *KAT1* expression levels in *KAT1OX*. *KAT1* expression in 3-day-old SD-grown Col-0 and *KAT1OX* seedlings at ZT0. Data are the means ± SE of biological triplicates (n = 3). Statistical difference relative to Col-0 is indicated by an asterisk (t-test; *, P < 0.05). The precise *P* value is provided in the Source Data file.

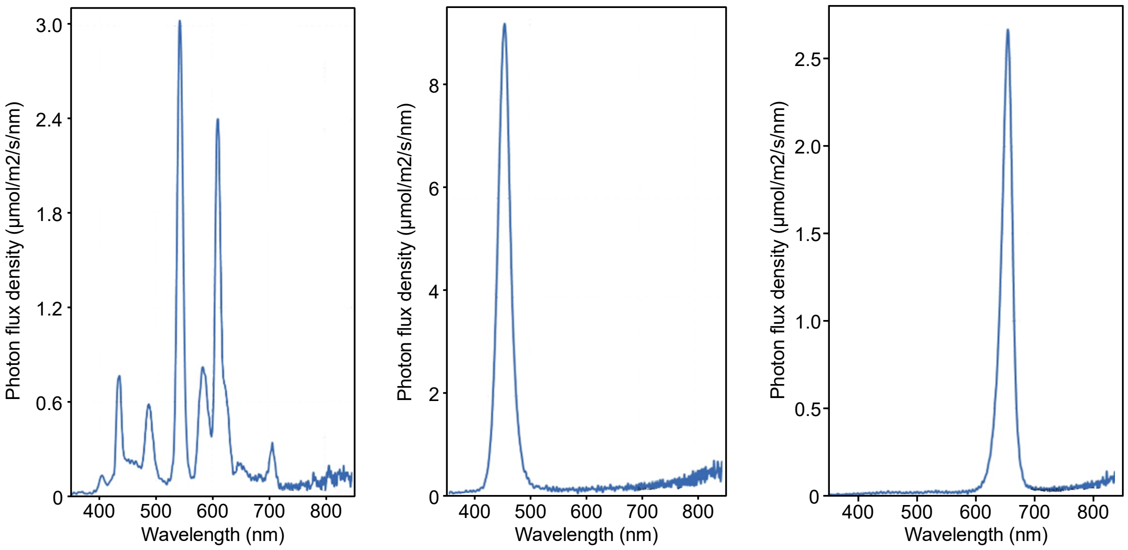

Supplementary Figure 16. Light spectra for the white, blue and red light sources. Light spectra of the white (left), blue (middle), and red (right) light sources used (see Methods for details).

Supplementary Table 1: Primers used for qRT-PCR.

For each gene analyzed by qRT-PCR the pair of primer sequences is shown. Columns indicate the primer name, the sequence, and the gene amplified. For primers described elsewhere, the reference is indicated.

Primer	Sequence	Gene
EMP1123	GCAATAAGGTACCTTTCGAC	*KAT1*
EMP1124	AAGCCTTGCAAATAGCGAGC	*KAT1*
EMP338	TATCGGATGACGATTCTTCGT	*PP2A*[80]
EMP339	GCTTGGTCGACTATCGGAATG	*PP2A*[80]

Supplementary Table 2: Primers used for ChIP-qPCR.

For each binding region analyzed by ChIP-qPCR the pair of primer sequences is shown. Columns indicate the primer name, the sequence, the gene amplified, and the binding region in the promoter (P1 and P2) or the gene body (P3).

Primer	Sequence	Gene	Binding region
EMP1152	GCATGGGAAGTGAAACTCTAAG	*KAT1*	P1
EMP1153	CGAGTGAGAAGAGAGTTTGGG	*KAT1*	P1
EMP1154	GCAAGCAATATGTCTTTGTTG	*KAT1*	P2
EMP1155	CCGACGGGAATGAGAAGTATG	*KAT1*	P2
EMP1178	CCAAACTTCTCACTTGCAAGTC	*KAT1*	P3
EMP1179	GATCCATATTGCAGCTCAAGC	*KAT1*	P3

Supplementary Table 3: Oligonucleotides used for Y1H.

Columns indicate the name, sequence, wild-type (WT) or mutated (Mut) G-box/PBE sequence, and the binding region in the promoter of *KAT1* (P1 or P2) (Fig. 5a).

Name	Sequence	G-box/PBE	Binding region
EMP1726	CCGGAAATCAAGTGTACA**CACATG**AAAAAACCCAC**CACGTG**AAGATCTCCTATT	WT	P1
EMP1727	CTAGAATAGGAGATCTT**CACGTG**GTGGGTTTTTT**CATGTG**TGTACACTTGATTT	WT	P1
EMP1760	CCGGAAATCAAGTGTACA**CcCATG**AAAAAACCCAC**CcCGTG**AAGATCTCCTATT	Mut	P1
EMP1761	CTAGAATAGGAGATCTT**CACGgG**GTGGGTTTTTT**CATGgG**TGTACACTTGATTT	Mut	P1

EMP1762	CCGGCATTCGCTACCTAGATTCGGTTTATC**CACGTG**GAGAGTTCACGTAGCTTC	WT	P2
EMP1763	CTAGGAAGCTACGTGAACTCTC**CACGTG**GATAAACCGAATCTAGGTAGCGAATG	WT	P2
EMP1764	CCGGCATTCGCTACCTAGATTCGGTTTATC**CcCGTG**GAGAGTTCACGTAGCTTC	Mut	P2
EMP1765	CTAGGAAGCTACGTGAACTCTC**CACGgG**GATAAACCGAATCTAGGTAGCGAATG	Mut	P2
