# Supplementary material for: PIF transcriptional regulators are required for rhythmic stomatal movements
Source: Nat Commun. 2024 May 29;15:4540. doi: 10.1038/s41467-024-48669-4 (PMC11137129; doi:10.1038/s41467-024-48669-4)
Supplement: Supplementary file 3 — Description of Additional Supplementary Files [file 41467_2024_48669_MOESM3_ESM.pdf]

Description of Additional Supplementary Files:

Supplementary Data 1: Genes regulated by PIFs and ABA in guard cells.

This is an excel file with the list of genes regulated by PIFs and ABA in guard cells, based on previous articles.
