# Supplementary figures and images for: PIF transcriptional regulators are required for rhythmic stomatal movements
Source: Nat Commun. 2024 May 29;15:4540. doi: 10.1038/s41467-024-48669-4 (PMC11137129; doi:10.1038/s41467-024-48669-4)

*pKAT1*  
Region P1

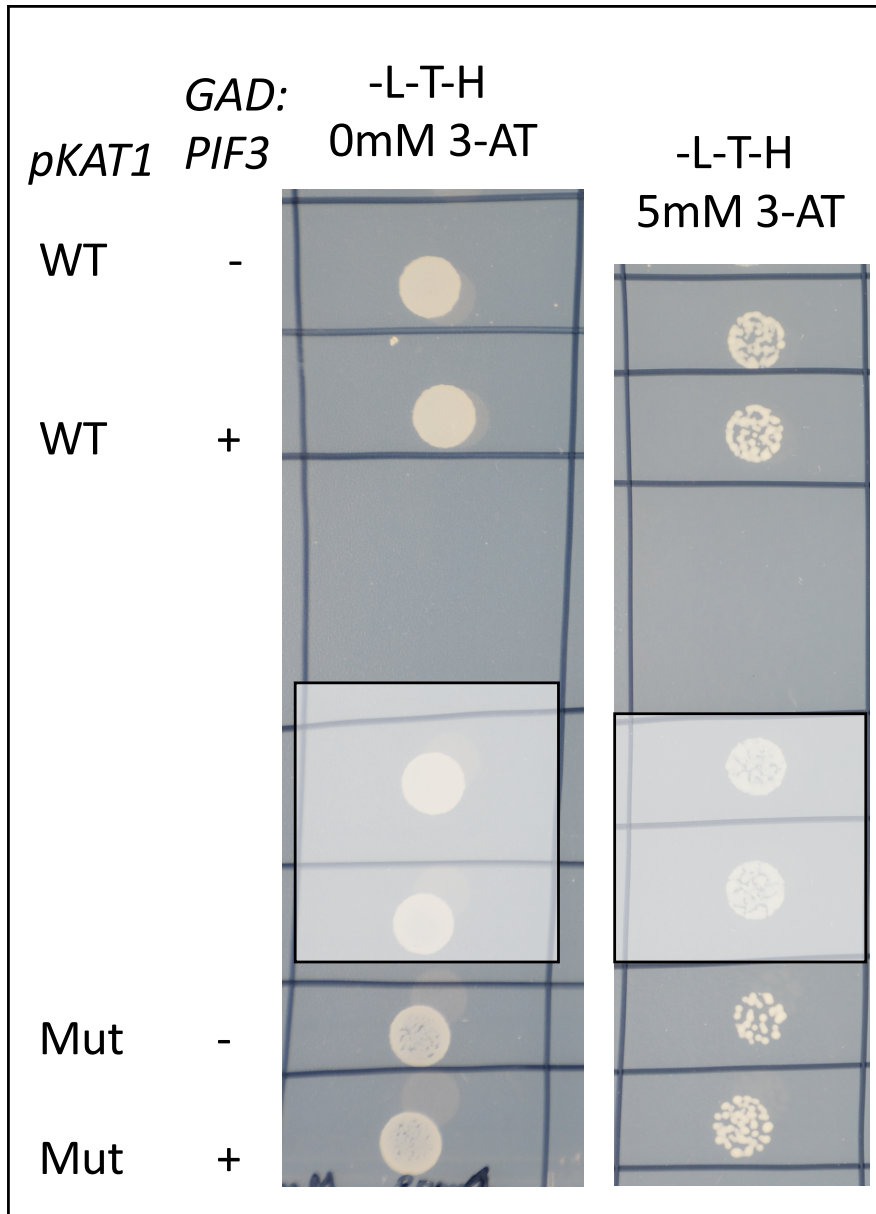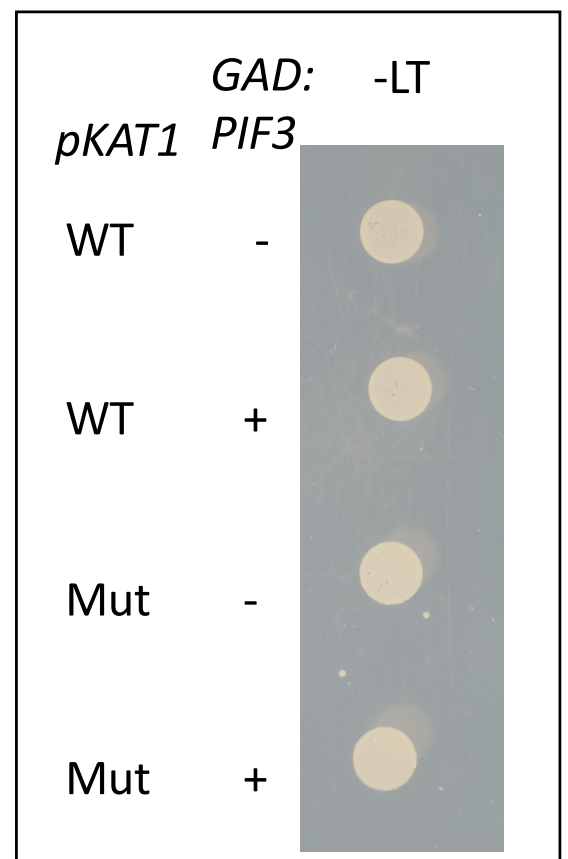

*pKAT1*  
Region P2

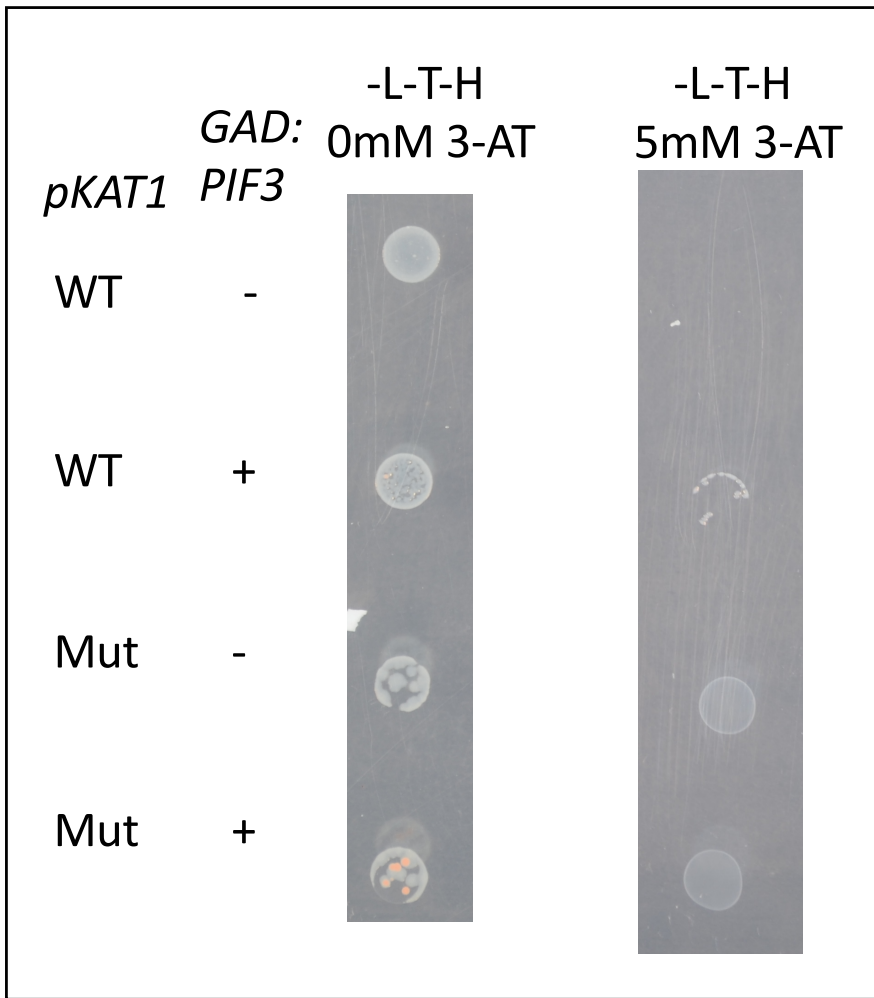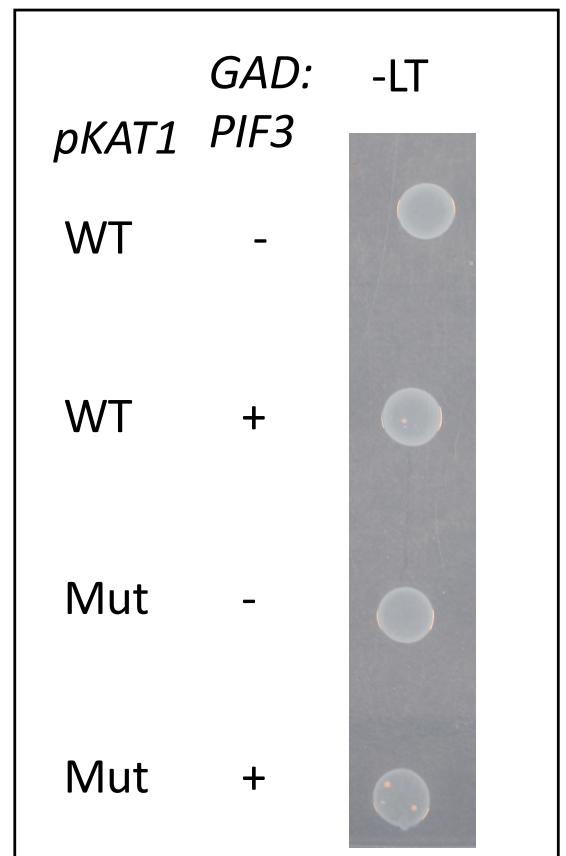

Supplement: Supplementary file 6 — Source Data Files [file 41467_2024_48669_MOESM6_ESM.zip › Supplementary Figure 10.pdf]
